# Supplementary material for: Overexpression of the Melatonin Synthesis-Related Gene SlCOMT1 Improves the Resistance of Tomato to Salt Stress
Source: Molecules. 2019 Apr 17;24(8):1514. doi: 10.3390/molecules24081514 (PMC6515010; doi:10.3390/molecules24081514)
Supplement: Supplementary file 1 [file molecules-24-01514-s001.pdf]

molecules

Article

Overexpression of the Melatonin Synthesis-Related Gene *SlCOMT1* Improves the Resistance of Tomato to Salt Stress

Dan-Dan Liu [1,†], Xiao-Shuai Sun [1,†], Lin Liu [1], Hong-Di Shi [1], Sui-Yun Chen [2,3,4,*] and Da-Ke Zhao [2,3,4,*]

[1] School of Agriculture, Yunnan University, Kunming, Yunnan 650091, China; liudandan@ynu.edu.cn (D.-D.L.); sunxiaoshuai2019@163.com (X.-S.S.); qiuqiu12-09@163.com (L.L.); dong19850412@163.com (H.-D.S.)
[2] Biocontrol Engineering Research Center of Plant Disease & Pest, Yunnan University, Kunming 650504, China;
[3] Biocontrol Engineering Research Center of Crop Disease & Pest, Yunnan University, Kunming 650504, China;
[4] School of Life Science, Yunnan University, Kunming 650504, China
* Correspondence: chensuiyun@ynu.edu.cn (S.-Y.C.); zhaodk2012@ynu.edu.cn (D.-K.Z.)
† These authors contributed equally to this work.

Supplementary Materials List

Supplementary Figure S1. Molecular cloning of *SlCOMT1*.

Supplementary Figure S2. Identification of transgenic tomatoes.

Supplementary Figure S3. *SlCOMT1* transgenic callus initiation, shoot regeneration, rooting and hardening of transgenic plants.

Supplementary Table S1. Primers used in this study.

Supplementary Table S2. PCR reaction system for cloning *SlCOMT1.*

Supplementary Table S3. PCR reaction procedure for cloning *SlCOMT1*.

Supplementary Table S4. Reaction system of quantification RT-PCR.

Supplementary Table S5. Configuration for tomato pre-culture medium.

Supplementary Table S6. Configuration for tomato differentiation medium.

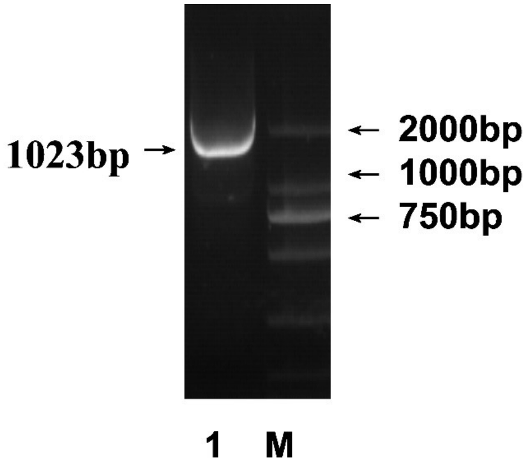

Supplementary Figure S1. Molecular cloning of *SlCOMT1*. 1, *SlCOMT1*; M, DNA Marker (DL2000).

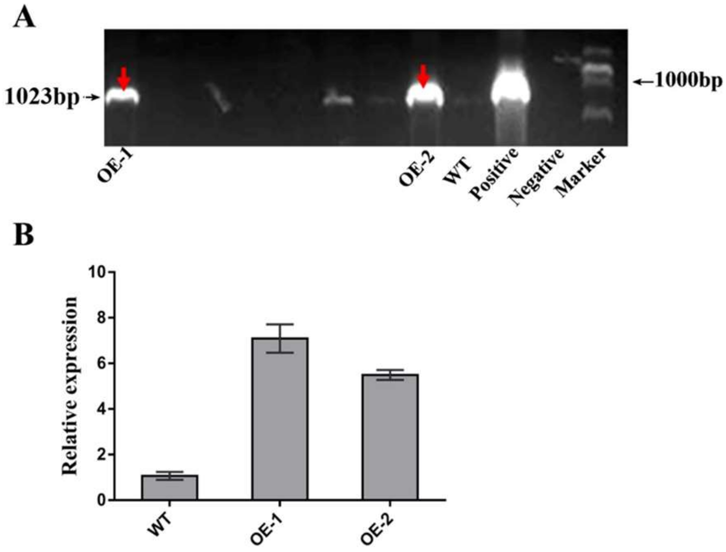

Supplementary Figure S2. Identification of *SlCOMT1* transgenic tomatoes. (**A**) Tomato cDNA from leaves were used for amplification of *SlCOMT1*; plasmid DNA of *SlCOMT1*-pMD19-T was used positive control, and ddH_2O as negative control. (**B**) *SlCOMT1* expression levels were detected using wild-type and transgenic tomato leaves. bp, base pair; WT, wild-type; OE, overexpression transgenic tomato.

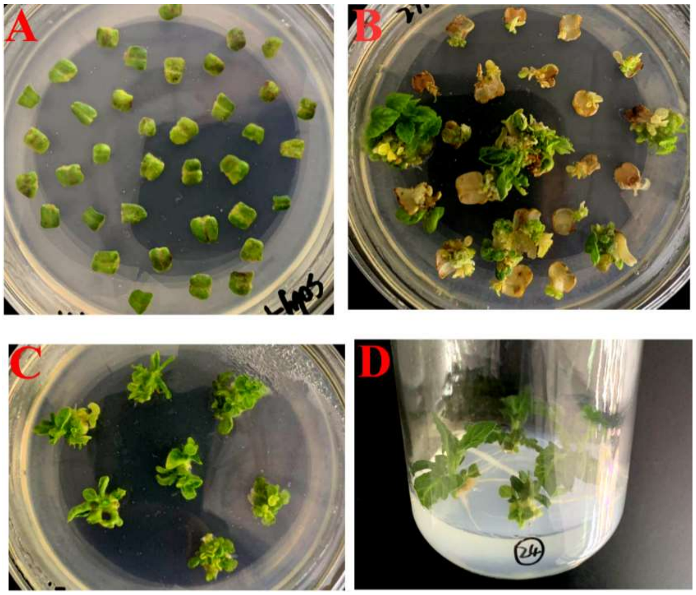

Supplementary Figure S3. Genetical transformation for *SlCOMT1* in tomato. (**A**) Cutting cotyledons on pre-cultured medium. (**B**) Callus initiation and shoot regeneration. (**C**) Regeneration seedling. (**D**) Rooting of transgenic plants.

Supplementary Table S1. Primers used in this study.

Gene	Forward Primers	Reverse Primers
SlCOMT1 (Molecular Cloning)	ATGCAACTGGCGAGTGCC	CTAGAGATTCTTGGTGAA
SlCOMT1 (RT-PCR)	GAATGCCGATGGTGTTTC	TGATGGATAAGGTGGTGTGA
SlCOMT1 (Transgenic plants)	AATGCAACTGGCGAGTGCC	CTAGAGATTCTTGGTGAA
SlCOMT1 (Protein induction)	GGATCCATGCAACTGGCGAGTG CC	GTCGACAGAGATTCTTGGTG AATTCCA
SlCOMT1 (Subcellular localization)	AAGGAAGCCCTTCACCGGTTCAA CAAGCCTAACTCAA	GGCGCGCCCACCCTTCTTGG TGAATTCCATAATCCAA

Supplementary Table S2. PCR reaction system for cloning *SlCOMT1.*

Component	Volume	
cDNA	Variable	As required
2.5mM dNTPs	4 μL	0.2 μM
TransTaq®DNA Polymerase	0.5 μL	2.5 units
10× TransTaq®HiFi Buffer I	5 μL	1×
Forward primers	1 μL	0.2 μM
Reverse primers	1 μL	0.2 μM
ddH_2O	Variable	-
Total	50 μL	-

Supplementary Table S3. PCR reaction procedure for cloning *SlCOMT1.*

Temperature	Time	
94 °C	5 min	
94 °C	30 s	35cycles
56 °C	30 s	
72 °C	45 s	
72 °C	10 min	

Supplementary Table S4. Reaction system of RT-PCR.

Reacion Reagents	Volume
2× UltraSYBR Mixture	10.0 μL
Foward Primer (10 μmol/L)	1.0 μL
Reverse Ptimer (10 μmol/L)	1.0 μL
cDNA	1.0 μL
ddH_2O	7.0 μL
Total	20 μL

Supplementary Table S5. Configuration for tomato pre-cultured medium.

MS Media (Free of Agar and Sucrose)	4.74 g
Sucrose	30 g
Indoleacetic acid (0.1 mg/mL)	5 mL
Zeatin (1 mg/mL)	2 mL
Agar power	8 g
pH	5.8
H_2O	Up to 1000 mL

Supplementary Table S6. Configuration for tomato differentiate medium.

**MS (Free of Agar and Sucrose)**	**4.74g**
Sucrose	30 g
Indoleacetic acid (0.1 mg/mL)	5 mL
Zeatin (1 mg/mL)	2 mL
Agar powder	8 g
Cephalosporin	1 mL
pH	5.8
H_2O	Up to1000 mL
